# The neutrophil-to-lymphocyte ratio as a prognostic biomarker in Guillain-Barre syndrome: a systematic review with meta-analysis

**DOI:** 10.3389/fneur.2023.1153690

**Published:** 2023-06-02

**Authors:** Miguel Cabanillas-Lazo, Carlos Quispe-Vicuña, Claudia Cruzalegui-Bazán, Milagros Pascual-Guevara, Nicanor Mori-Quispe, Carlos Alva-Diaz

**Affiliations:** ^1^Red de Eficacia Clínica y Sanitaria (REDECS), Lima, Peru; ^2^Sociedad Científica de San Fernando, Lima, Peru; ^3^Facultad de Medicina, Universidad Nacional Mayor de San Marcos, Lima, Peru; ^4^Servicio de Neurología, Departament de Medicina y Oficina de Apoyo a la Docencia e Investigación (OADI), Hospital Daniel Alcides Carrión, Callao, Peru; ^5^Universidad Señor de Sipán, Chiclayo, Peru

**Keywords:** Guillain-Barre syndrome, NLR, neutrophil to lymphocyte ratio, diagnosis, prognosis

## Abstract

**Background and objectives:**

Guillain-Barre syndrome (GBS) is an immune-mediated neuropathy. This has raised the possibility that the neutrophil-lymphocyte ratio (NLR) may be a biomarker of its activity. We conducted a systematic review and meta-analysis to summarize the evidence of NLR as a potential biomarker for GBS.

**Methods:**

We systematically searched databases (PubMed, Ovid-Medline, Embase, Scopus, Web of Science, SciELO Citation Index, LILACS, and Google Scholar) until October 2021 for studies evaluating pre-treatment NLR values in GBS patients. A meta-analysis using a random-effects model to estimate pooled effects was realized for each outcome and a narrative synthesis when this was not possible. Subgroup and sensitivity analysis were realized. GRADE criteria were used to identify the certainty of evidence for each result.

**Results:**

Ten studies from 745 originally included were selected. Regarding GBS patients versus healthy controls, a meta-analysis of six studies (968 patients) demonstrated a significant increase in NLR values in GBS patients (MD: 1.76; 95% CI: 1.29, 2.24; I2 = 86%) with moderate certainty due to heterogeneity of GBS diagnosis criteria used. Regarding GBS prognosis, assessed by Hughes Score ≥ 3, NLR had a sensitivity between 67.3 and 81.5 and a specificity between 67.3 and 87.5 with low certainty due to imprecision, and heterogeneity. In relation to respiratory failure, NLR had a sensitivity of 86.5 and specificity of 68.2 with high and moderate certainty, respectively.

**Discussion:**

With moderate certainty, mean NLR is higher in GBS patients compared to healthy controls. Furthermore, we found that NLR could be a prognostic factor for disability and respiratory failure with low and moderate certainty, respectively. These results may prove useful for NLR in GBS patients; however, further research is needed.

**Systematic review registration:**

https://www.crd.york.ac.uk/PROSPERO/, identifier: CRD42021285212.

## 1. Introduction

Guillain-Barre syndrome (GBS), an immune-mediated peripheral neuropathy, is the most common cause of acute flaccid paralysis and is characterized by rapidly progressive weakness or sensory loss usually followed by slow clinical recovery (1). The annual global incidence rate is 1–2 cases per 100,000 people per year (2). The global prevalence of GBS has shown an increase of 6.4% from 1990 to 2019, reporting in the latter year, more than 150,000 cases and more than 44 000 years lived with disability (YLD) (3). Also, Asia and Central and South America are the regions with the highest frequency (30–65%) of the axonal subtype of GBS which is the most severe subtype of the disease (4). In addition, death or severe disability results in almost 20% of patients despite adequate treatment (5).

The most frequent risk factor of GBS is gastrointestinal and respiratory infection (6), which generates a cellular and humoral response by T and B cells. The latter induces an antiganglioside antibody response that crosses the blood-nerve barrier and activate the complement. Likewise, T-cell activation leads to cytokine release, Schwann cell damage and myelin destruction (7). This mechanism results in axonal degeneration of motor and sensory fibers of cranial and peripheral nerves (1). This is known as “molecular mimicry autoimmunity.”

Despite that the role of the neutrophil in GBS pathophysiology is not well understood, several investigations suggest the neutrophil-to-lymphocyte ratio (NLR) as a possible index of immune function, since the systemic inflammation generated by the disease usually results in neutrophilia and lymphocytopenia (1, 6–8).

Although the diagnosis of GBS is mainly based on a combination of clinical criteria, cerebrospinal fluid (CSF) analysis and nerve conduction studies (Brighton criteria); some criteria are not present in early stages of the disease, such as cytological albumin dissolution (after the first week) or alterations in neuroconduction studies (after the second week) (9). This has led to the search for biomarkers to establish a rapid and accurate diagnosis, using serum, peripheral nervous tissue and CSF as the main sources.

Despite all this, there is a lack of accessible and reliable biomarkers of systemic inflammation in neurology, which could provide information to differentiate or predict GBS activity (10–12). The neutrophil-to-lymphocyte ratio (NLR) is calculated from the white blood cell count and is a new biomarker used in neurological diseases such as autoimmume encephalitis and multiple sclerosis (8, 13). Even now, it's part of routine blood tests and is therefore available for research as possible alternatives. So, the importance of NLR lies of its performance, its low cost, its availability in routine examinations and its easy acceptance by physicians and patients.

NLR is more reflective of systemic inflammation compared to other leukocyte subtypes, is easier to obtain by blood test, and is stable and reliable (14). Given that the prognosis of GBS is not fully elucidated, NLR could be an accessible prognostic factor of GBS activity and severity. For those reasons, this systematic review summarizes current knowledge about the potential of NLR as a biomarker in GBS.

## 2. Methods

This systematic review was reported according to the Preferred Reporting Items for Systematic Reviews and Meta-Analyses (PRISMA) (15). The study protocol was registered in PROSPERO with the code CRD42021285212.

### 2.1. Data sources

We searched in PubMed, Ovid-Medline, Embase, Scopus, Web of Science, SciELO Citation Index, LILACS and Google Scholar until October 2021. The search strategy is detailed in Supplementary Table 1. There were no restrictions on language or publication date. We completed the search by reviewing the bibliographic references of the included studies and selecting the articles that met the requirements.

### 2.2. Eligibility criteria

Studies were included if they met the following criteria: (1) Analytical observational studies (cross-sectional, case-control and cohort studies); (2) Studies included adult participants (aged > 18 years old) and (3) NLR values assessed at pre-treatment period in GBS patients. We excluded narrative and systematic reviews, studies in non-humans, case reports, conference abstracts and letters.

### 2.3. Study selection

Results from electronic searches were exported to Endnote X9 and duplicate documents were removed. After, two reviewers (CCB and MPG) performed a peer review process using Rayyan QCRI (https://rayyan.qcri.org/) for the selection of articles according to the inclusion criteria and then reading the full text. Any discrepancies were resolved by consensus or a third author (CAD). The complete list of excluded articles is provided in Supplementary Table 2.

### 2.4. Outcomes

Our outcomes were (1) Assessment of NLR comparing GBS patients with healthy control subjects (NLR is the biochemical parameter calculated by dividing the absolute neutrophil count by the absolute lymphocyte count); and (2) Assessment of NLR comparing GBS mild and severe, which was assessed with at least one of the following from discharge: (a) Hughes's score (16) using a scale from 0 to 6 ranging from no symptoms to death, (b) Medical Research Council (MRC) scale (17) using a scale from 0 to 5 ranging from no visible contraction to active movement against full resistance, or (c) respiratory failure.

### 2.5. Data extraction

Two authors (CCB and MPG) independently carried out information using a data extraction form and any disagreements were resolved by consensus and ultimately a third author (CAD). We extracted the following information: title of the study, first author, year of publication, study design, country where the study was performed, number of participants, sex, age, sample time, criteria for diagnosis of GBS, mean or median NLR according to sample stratification, follow-up crude and adjusted association measures, type of outcome and its definition. We contacted the corresponding author through email if additional data was needed.

### 2.6. Risk of bias assessment

The quality of the studies was assessed with the Newcastle Ottawa Scale (18) by two authors (MCL and CQV) and any discrepancies were resolved by consensus. This tool evaluates the quality of published observational studies and is based on three items: selection, comparability, and outcome/exposure. Each item has sub-items, on which a star-based score was assigned. Studies with scores ≥ 6 were considered as having a low risk of bias (high quality), scores of 4–5 as having a moderate risk of bias, and scores < 4 as having a high risk of bias (19). If the studies had a double design (cases and controls, cohorts), the design was considered according to the main objective of the study.

### 2.7. Statistical analysis

A meta-analysis was planned for each outcome; however, when this was not possible due to unavailable data, narrative synthesis was performed. Meta-analyses were performed using the inverse variance method and the randomized effects model. The variance between studies (τ2) was estimated using the DerSimonian-Laird method. Mean differences (MD) with their 95% confidence intervals (CI 95%) were pooled if the studies had a confounder control. Heterogeneity between studies was assessed using the *I*^2^ statistic. Heterogeneity was defined as low if *I*^2^ < 30%, moderate if *I*^2^ = 30–60%, and high if *I*^2^ > 60%. The metacont function of the meta-package in R 4.1.0 was used (www.r-project.org).

We performed subgroup analyses by reported diagnostic criteria. Finally, we performed a leave-one-out sensitivity analysis, and other only including studies with case-control matches.

### 2.8. Evidence certainty assessment

Two authors (CQV and CCB) assessed the certainty of our pooled results and narrative synthesis applying the Grading of Recommendation, Assessment, Development, and Evaluation (GRADE) on continuous outcomes (29) and narrative synthesis (30), respectively. This assessment is based on five domains: study limitations (risk of bias of the studies included), imprecision (sample size and confidence interval), indirectness (generalizability), inconsistency (heterogeneity), and publication bias as stated in the GRADE handbook (31) and prognostic factor (32). We adapted the assessment to our results. The certainty of the evidence was characterized as high, moderate, low, or very low.

### 2.9. Ethical considerations

This systematic review included published and open information in which no human subjects participated. Thus, no ethics committee approval was required.

## 3. Results

### 3.1. Study selection

**Figure 1:**
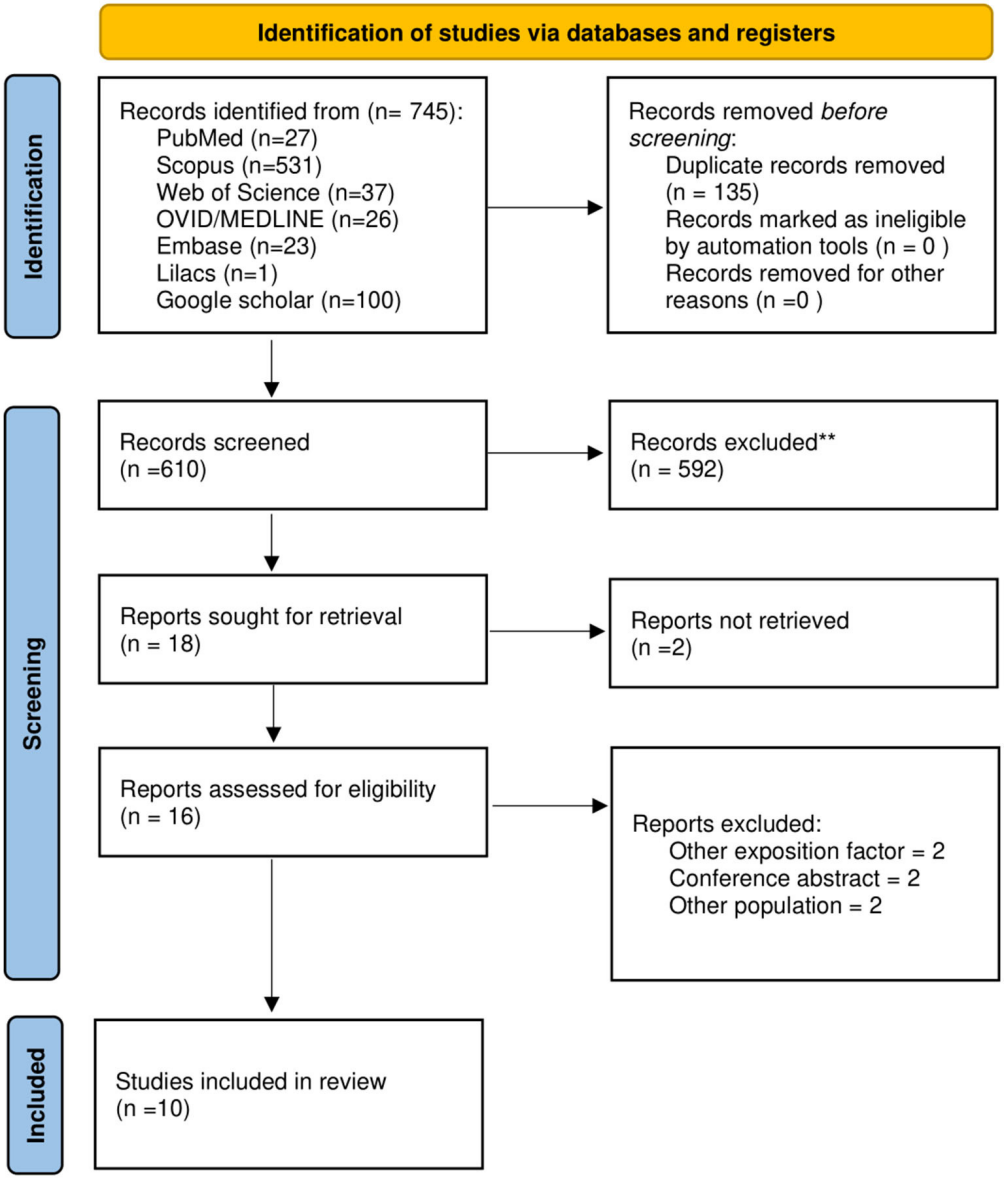
PRISMA flowchart of included studies.

We identified 745 studies through our systematic search. We removed duplicated and screened 610. Finally, 10 articles (6, 20–28) were included (Figure 1).

### 3.2. Characteristics of studies

Five studies (21–25) included both cohort and case-control designs, four (6, 26–28) included only retrospective cohorts and one (20) was a case-control study. The total number of participants was 1,626 (1,055 GBS patients and 571 healthy controls). The average age range of GBS patients was between 32.3 and 55.0. The studies characteristics are summarized in Table 1.

**Table 1:** Characteristics of included studies evaluating the clinical significance of neutrophil-to-lymphocyte ratio (NLR) in Guillain-Barré syndrome (GBS) (*n* = 10).

**Study-ID**	**Country**	**Study design**	**GBS diagnostic criteria**	**N GBS**	**N Controls**	**Match GBS-control**	**Characteristics of the GBS population: age (years) mean**	**Blood sample time**	**Follow-up**
Bedel et al. (20)	Turkey	Case-control	Asbury (1990)	98	101	NR	Male (%): 63.3 Age: 55.02 (17.4)^*^	Admission	NR
Ethemoglu and Calik (21)	Turkey	Retrospective cohort and Case-control	NR	68	63	Age	Male (%): NR Age: 48.5 (19.0)^*^	Day post admission	3 months
Geyik et al. (22)	Turkey	Retrospective cohort and Case-control	Asbury (1990)	94	101	Age Gender	Male (%): 66 Age: 48.7 (20.6)^*^	Admission	1 month
Hashim et al. (23)	Egypt	Prospective Cohort and Case-control	Asbury (1990)	35	40	Age Gender	Male (%): 42.9 Age: 32.29 (13.4)^*^	Admission	1 month
Huang et al. (24)	China	Retrospective cohort and Case-control	Dutch Neuromuscular Research Support consensus (2001)	117	217	Age Gender	Male (%): 62.4 Age: 49.36 (16.9)^*^	Day post admission	2 months
Gümüşyayla and Vural (25)	Turkey	Retrospective cohort and Case-control	NR	50	49	NR	Male (%): NR Age: 52.8 (17.0)^*^	Day post admission	3 months
Sahin et al. (26)	Turkey	Retrospective cohort	Asbury (1990)	24	NR	NR	Male (%): NR Age: 41 (16.0)^*^	Admission	6 months
Tunç (27)	Turkey	Retrospective cohort	Asbury (1990)	81	NR	NR	Male (%): 54.3 Age: 52.3 (18.4)^*^	Day post admission	1 month
Ning et al. (28)	China	Retrospective cohort	Asbury (1990)	426	NR	NR	Male (%): 59.6 Age: 49.5 (4.7)^*^	Admission	NR
Ozdemir (6)	Turkey	Retrospective cohort	Brighton (2011)	62	NR	NR	Male (%): 58.1 Age: 48.0 (19.9)^*^	Admission	Discharge

^*^Mean (standard deviation). NR, not reported.

### 3.3. GBS patients and healthy controls

**Figure 2:**
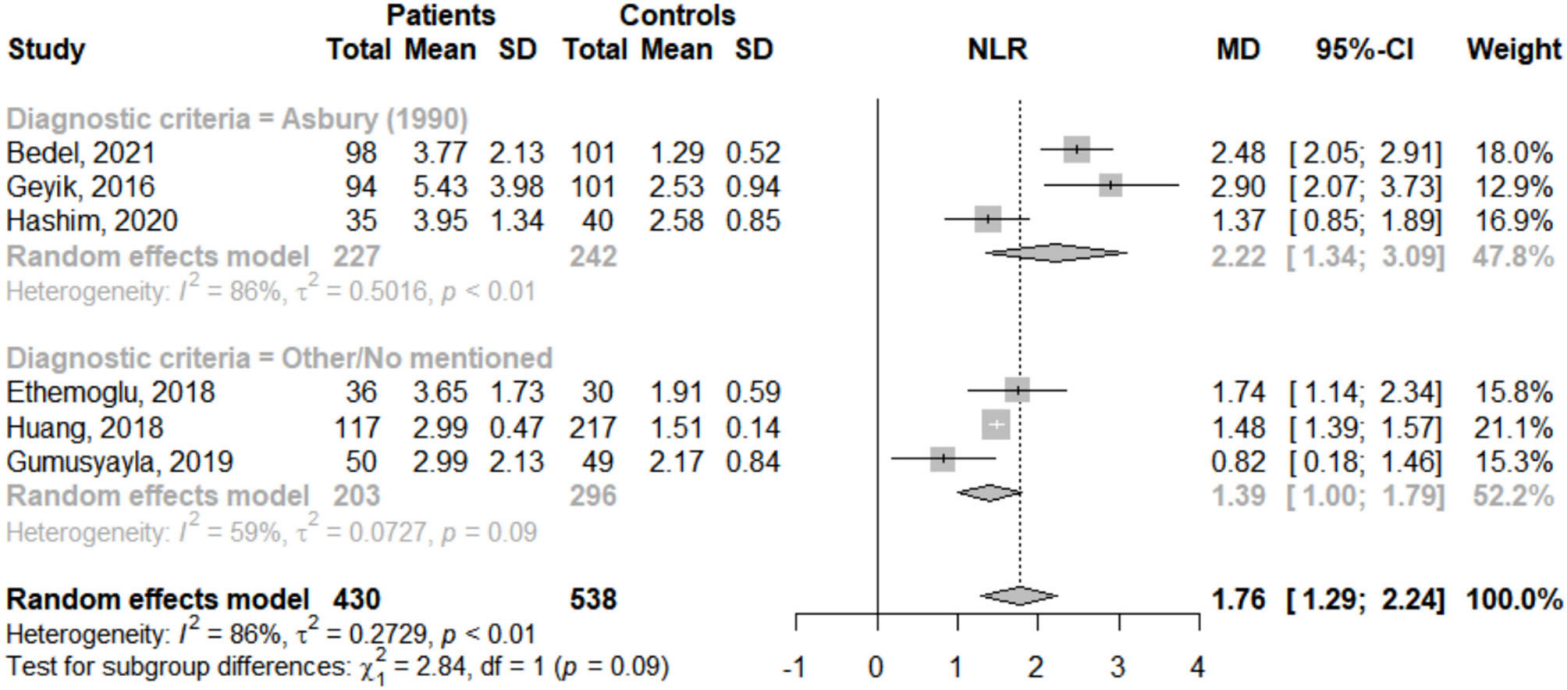
Mean difference of neutrophil-to-lymphocyte ratio (NLR) between GBS patients and healthy controls according to reported diagnostic criteria.

Among the ten studies that met our inclusion criteria, six (20–25) were pooled. A total of 968 participants were selected, of which 430 were assigned to the GBS group and 538 to the control group. In the pooled analysis, a significant increase in NLR was observed in GBS vs. control groups but with high heterogeneity (MD: 1.76; 95% CI: 1.29; 2.24; *I*^2^ = 86%) (Figure 2).

To identify potential sources of heterogeneity, subgroup analysis was performed according to reported diagnostic criteria. NLR was significantly higher in GBS patients compared to healthy controls regardless of whether GBS was diagnosed with Asbury criteria (MD: 2.22; 95% CI: 1.34; 3.09; *I*^2^ = 86%) or other/no mentioned (MD: 1.39; 95% CI: 1.00; 1.79; *I*^2^ = 59%) (Figure 2). In addition, heterogeneity was high among studies that used the Asbury criteria and moderate in those that used other/no mentioned.

Regarding sensitive analysis, when single studies were sequentially removed, no significant effect on the pooled MD was observed, with an effect size ranging from 1.59 to 1.94 and *I*^2^ ranged from 76 to 89 (Supplementary Table 3). Additionally, when studies without case-control match were excluded, no significant effect was described (MD: 1.75; 95% CI: 1.29; 2.21; *I*^2^ = 75%). These suggest that the results estimated by meta-analysis were stable (Supplementary Table 4).

### 3.4. GBS mild and severe

Nine studies (6, 21–28) (925 GBS patients) assessed NLR as a poor prognostic factor. Only three reported sensitivity and specificity. Regarding prognosis of disability (Hughes ≥ 3), Hashim et al. (23) included 35 patients whose cut-off point was 4.4 with a sensitivity of 81.5% and specificity of 87.5%, Huang et al. (24) included 117 participants whose cut-off point was 3.05 with a sensitivity of 67.3% and specificity of 67.3% (Table 2).

**Table 2:** Characteristics of included studies evaluating the use of neutrophil-to-lymphocyte ratio (NLR) as a prognosis factor of Guillain-Barré syndrome (GBS) patients (*n* = 9).

**Study-ID**	**Number of analized patients**	**Outcome**	**NLR mean (SD) in poor outcome**	**NLR mean (SD) in good outcome**	**Correlation NLR-Hughes**	**NLR cutoff**	**Sensitivity (%)**	**Specificity (%)**	**Area under the curve (AUC)**	**Follow-up**
Geyik et al. (22)	94	Response to plasmapheresis^†^	9.10 (3.89)	4.67 (3.57)	0.36^*^	NR	NR	NR	NR	1 month after discharge
Hashim et al. (23)	35	Hughes score ≥ 3	5.11 (1.78)	3.60 (1.18)	0.55^*^	4.40	81.5	87.5	0.85	1 month after admission
Huang et al. (24)	117	Hughes score ≥ 3	3.37 (0.83)	2.34 (0.45)	NR	3.05	67.3	67.3	0.72	2 months after admission
Ethemoglu and Calik (21)	36	Hughes score ≥3	2.92 (1.69)	4.38 (1.49)	NR	NR	NR	NR	NR	At discharge
Gümüşyayla and Vural (25)	50	Hughes score	NR	NR	0.36^**^	NR	NR	NR	NR	3 months after admission
Tunç (27)	81	Hughes score	NR	NR	0.23^*^	NR	NR	NR	NR	1 month after admission
Ning et al. (28)	426	Respiratory failure	6.69 (1.19)	2.70 (0.43)	NR	3.5	86.5	68.2	0.79	NR
Sahin et al. (26)	24	MRC sum score	NR	NR	0.007^¥^^**^	NR	NR	NR	NR	6 months after admission
Ozdemir (6)	62	Hughes score	NR	NR	NR^††^	NR	NR	NR	NR	At discharge

SD, Standard deviation; NR, Not reported; MRC, Medical Research Council. ^*^Pearson r, ^**^Spearman rho. ^†^Outcome not specified; ^††^Correlation r no specified but described as not significant;^¥^Correlation between NLR and MRC2st score.

### 3.5. Respiratory failure

For the respiratory failure outcome, only one large-size study reported this. Ning et al. (28) included 426 patients whose cut-off point was 3.5 with a sensitivity of 86.5% and a specificity of 68.2% (Table 2).

### 3.6. Risk-of-bias assessment

We performed the risk of bias of the ten included studies. Eight articles had “low risk of bias.” Among the cohort studies, Gümüşyayla et al. (25) scored the lowest for deficiencies in the comparability domain, while Ning et al. (28), Huang et al. (24), and Ethemoglu et al. (21) scored the highest. The only case-control design considered for risk of bias assessment (20) scored 6/9 for deficiencies in information response rate and the selection of controls (Supplementary Table 5).

### 3.7. Evidence certainty

Regarding GBS patients and healthy controls, we judged the certainty of the included evidence as moderate. We started the evaluation from high certainty because we included observational studies (comparative observational design). We downgraded according to the high heterogeneity between the studies (*I*^2^ = 86%) (Table 3). About the severity and respiratory failure outcome, we started the assessment from high certainty because all included studies were observational studies (cohort design). For severity, Hughes's score ≥ 3 had a low certainty for sensitivity and specificity, downgraded according to inconsistency due to high heterogeneity (studies showed a sensitivity of 67.3 to 81.5 and specificity of 67.3 to 87.5) and imprecision (sensitivity and specificity crossed through the imprecision point of 70%). For respiratory failure, was assessed as moderate and high certainty to specificity and sensitivity, respectively, downgraded according to imprecision (specificity crossed through the imprecision point of 70%).

**Table 3:** Summary of findings.

**Outcome**	**No. of participants (studies)**	**Certainty of the evidence (GRADE)**	**Anticipated absolute effects**	**95% CI**
			**Mean difference (MD)**	
Mean difference between GBS patients and healthy participants	968 (6 observational studies)	⊕⊕⊕○ Moderate^a^	1.76	1.29–2.24
Sensitivity for prognosis Hughes's score ≥ 3	152 (2 observational studies)	⊕⊕○○ Low^b, c^	The studies showed a sensitivity of 67.3 to 81.5 for prognosis in GBS patients.	
Specificity for prognosis Hughes's score ≥ 3	152 (2 observational studies)	⊕⊕○○ Low^b, c^	The studies showed a specificity of 67.3 to 87.5 for prognosis in GBS patients	
Sensitivity for prognosis Respiratory failure	426 (1 observational study)	⊕⊕⊕⊕ High	The study showed a sensitivity of 86.5 of the NLR for predicting respiratory failure in GBS patients.	
Specificity for prognosis Respiratory failure	426 (1 observational study)	⊕⊕⊕○ Moderate^c^	The study showed a specificity of 68.2 of the NLR for predicting respiratory failure in GBS patients.	

CI, confidence interval; MD, mean difference. GRADE Working Group grades of evidence: High certainty, we are very confident that the true effect lies close to that of the estimate of the effect. Moderate certainty, we are moderately confident in the effect estimate: the true effect is likely to be close to the estimate of the effect, but there is a possibility that it is substantially different. Low certainty, our confidence in the effect estimate is limited: the true effect may be substantially different from the estimate of the effect. Very low certainty, we have very little confidence in the effect estimate: the true effect is likely to be substantially different from the estimate of effect. ^a^High inconsistency was detected in meta-analyses. The calculated I^2^ was >60%. ^b^Inconsistency: values of studies are heterogeneous among themselves. ^c^Imprecision due to confidence interval < 70% in one study.

## 4. Discussion

### 4.1. Summary of main results

Our results revealed a higher mean NLR in GBS patients compared to healthy controls. Furthermore, we found that NLR could be a prognostic factor for disability and respiratory failure.

### 4.2. NLR in GBS patients vs. healthy controls

Our pooled analysis showed that mean NLR was significantly higher in GBS patients than in healthy patients. These results were not altered by any study or by studies reporting case-control matching as our sensitivity analysis demonstrates. This agrees with previous reviews that evaluated the NLR in other autoimmune diseases. Olsson et al. (8) evaluated 4 case-control studies and found that the NLR was higher in patients with MS than in healthy controls. Similarly, Paliogiannis et al. (33) reported that a meta-analysis of 12 studies showed a higher values NLR (SMD = 0.69, 95% CI 0.53–1.85, *p* < 0.001) in patients with psoriasis than in healthy patients. In turn, Erre et al. (34) and Ma et al. (35) reported similar results for rheumatoid arthritis (SMD = 0.79, 95% CI 0.55–1.03; *p* < 0.001) and systemic lupus erythematosus (SMD = 1.004, 95% CI = 0.781–1.227, *P* < 0.001), respectively. This would show that high NLR values could differentiate between autoimmune diseases and healthy persons. The biological mechanism that would explain these relationship between NLR and GBS, is the increase of immune cells such as neutrophils, macrophages, dendritic cells and T cells in response to peripheral nerve injury (36).

### 4.3. NLR as prognostic factor in GBS patients

Despite its importance and novelty, the presence of systematic reviews about NLR and its prognosis in neurological diseases, specifically autoimmune diseases, is limited. Olsson et al. (8) reported five studies that evaluated NLR as a prognostic factor in multiple sclerosis. Among these, one reported that high NLR (>3.9) was an independent predictor of disability progression (*p* = 0.001). Also, Guzel et al. (37) reported that a cut-off point of 4.52 (sensitivity: 96.1%; specificity: 57.1%) in multiple sclerosis patients have a discriminatory capacity for disability [Expanded Disability Status Scale (EDSS) ≥ 5]. These results were similar to our results in patients with GBS where the cut-off points were 4.40 (sensitivity: 81.5%; specificity: 87.5%) and 3.05 (sensitivity: 67.3%; specificity: 67.3%) for disability (Hughes ≥ 3). This is also supported by an observational study in which 34 patients with autoimmune encephalitis presented a cut-off value of 4.82 (sensitivity: 78%; specificity: 88%) to predict severity defined as Modified Rankin Scale (mRS) score > 3 (13). This can be explained by the presence of serum low-density neutrophils (LDN), which are immature and degranulated cells with immunomodulatory capacity that are prematurely mobilized from the bone marrow (38, 39). These LDNs secrete higher levels of proinflammatory cytokines, including interleukin 10 and interferon 21 (40). Levels of this cell type have been correlated with poor prognosis in patients with GBS (41). However, despite the findings, the pathological levels of NLR for different diseases have not yet been standardized, with a possible range of normality being between 0.78 and 3.53 (42).

Otherwise, one of our included studies also evaluated the ability of NLR to predict respiratory failure in patients with GBS with a cut-off point of 3.5 (sensitivity: 86.5%; specificity: 68.2%). According to Moisa et.al. (43), NLR with a cut-off point > 2 was found to predict the need for mechanical ventilation and >11 for mortality at 48 h after admission to ICU in patients with severe or critical pneumonia due to COVID-19. Nair et al. (44) also reported a cut-off point of 4.6 (sensitivity: 79.2%; specificity: 62.3%) for ventilatory assistance 24 h after admission to ICU, which is supported by an SR where the NLR cut-off points ranged from 3.3 to 5.9 predicted a severe COVID-19 condition defined as present respiratory failure (45). As mentioned above, the correlation between NLR and a worse prognosis in GBS could be because neutrophils are one of the first lines of response to peripheral nerve injury so it proliferates within the first hours of trauma (46). This would make the NLR more susceptible to any poor prognosis with respect to other biomarkers.

### 4.4. Recommendations for future research

According to GRADE approach, the certainty of evidence from the NLR was moderate in GBS patients vs. healthy controls due to high heterogeneity between studies. In our subgroup analysis by GBS diagnostic criteria, it was possible to observe differences in effect size and heterogeneity. This aspects should be expanded in future studies, in addition to taking into account the Brighton criteria (47), since only one study reported using them (6). Finally, we recommend conducting studies that use the NLR for the differential diagnosis of other polyneuropathies (chronic inflammatory demyelinating polyradiculoneuropathy, metabolic diseases, toxicity, others).

Certainty for sensitivity and specificity for poor prognosis (Hughes ≥ 3) was low due to heterogeneity and imprecision. Therefore, prospective studies with long-term follow-up are needed to determine optimal cut-off point taking into account the different prognosis of axonal and demyelinating GBS (48, 49). In addition, since majority of included studies was realized in one country (Turkey), it is recommended to analyze the NLR in different GBS populations due there are variations in clinical patterns and severity between Europe/America and Asia (50). On the other hand, the modified Erasmus GBS Outcome Score (mEGOS) is a validated tool that predicts short- and long-term disability with clinical variables that are easy to obtain at admission (51), so the NLR could be added to that tool for better prognostic accuracy. Regarding the prognosis of respiratory failure, the evidence was moderate based on a single large size cohort; however, there are no data about the time in which this failure will be established so more studies are recommended to evaluate the NLR as a prognostic of early requirement of mechanic ventilation. Like mEGOS, there is a validated tool that predicts respiratory failure (EGRIS) based on three simple variables (52, 53), so NLR could also be a biomarker to take into account due to its similar characteristics.

### 4.5. Clinical applicability

NLR is not only an indicator of systemic inflammation but can also serve as an early warning of a pathological state or process (54). For neurological diseases, it has demonstrated clinical utility in assessing the activity or prognosis of diseases such as multiple sclerosis (8). Our results show that the NLR was significantly higher in GBS patients with respect to healthy patients and that, in turn, it was also a poor prognostic factor. The early identification of GBS patients with poor prognosis could benefit them since they can be treated with different doses, although this is not yet conclusive (55, 56). In addition, determine who will have respiratory failure may allow early intubation and reduce the risk of early-onset pneumonia (57). Several biomarkers associated with GBS such as cerebrospinal lipids (58) or IL-8 have been reported to differentiate GBS from chronic inflammatory demyelinating polyneuropathy (CIDP) (59).However, the clinical importance of NLR lies in the simplicity of its performance, its low cost, its availability in routine examinations and its easy acceptance by physicians and patients. Therefore, if its role is confirmed with more and better studies, it should be recommended for its diagnostic and prognostic application.

### 4.6. Limitations and strengths

There are some limitations to this systematic review. First, most of the studies were retrospective in nature, so their results could be prone to confounding. In addition, most on the included studies were realized in only one country (Turkey) so it's necessary to analyze the NLR values of other GBS patients from different parts of the world, to seek for variations in clinical patterns and severity. Also, there was significant heterogeneity among the included articles in our meta-analysis. However, our results were strengthened by sensitivity analyzes performed. Our study also has strengths. This is the first systematic review that evaluates the clinical significance of NLR in the diagnosis and prognosis of GBS. Second, we conducted a comprehensive systematic search without language or time restrictions. Finally, we performed an assessment of the certainty of our results using the GRADE criteria.

### 4.7. Conclusions

Based on the evidence available, with moderate certainty, mean NLR is higher in GBS patients compared to healthy controls. Furthermore, with low and moderate certainty, we found that NLR could be a prognostic factor for disability and respiratory failure, respectively. Future prospective studies in different regions with long-term follow-up are required to determine optimal cut-off values considering the possibility of including NLR in validated prognostic tools.

## Data availability statement

The original contributions presented in the study are included in the article/Supplementary material, further inquiries can be directed to the corresponding author.

## Author contributions

MC-L: drafting/revision of the manuscript for content, including medical writing for content, study concept or design, and analysis or interpretation of data. CQ-V and CA-D: drafting/revision of the manuscript for content, including medical writing for content, major role in the acquisition of data, and study concept or design. CC-B, MP-G, and NM-Q: drafting/revision of the manuscript for content, including medical writing for content, and major role in the acquisition of data. All authors contributed to the article and approved the submitted version.

## References

[1] van den Berg B Walgaard C Drenthen J Fokke C Jacobs BC van Doorn PA. Guillain-Barré syndrome: pathogenesis, diagnosis, treatment and prognosis. Nat Rev Neurol. (2014) 10:469–82. 10.1038/nrneurol.2014.121 25023340

[2] Sejvar JJ Baughman AL Wise M Morgan OW. Population incidence of Guillain-Barré syndrome: a systematic review and meta-analysis. Neuroepidemiology. (2011) 36:123–33. 10.1159/000324710 21422765 PMC5703046

[3] Bragazzi NL Kolahi AA Nejadghaderi SA Lochner P Brigo F Naldi A . Global, regional, and national burden of Guillain-Barré syndrome and its underlying causes from 1990 to 2019. J Neuroinflammation. (2021) 18:264. 10.1186/s12974-021-02319-4 34763713 PMC8581128

[4] Kuwabara S Yuki N. Axonal guillain-barré syndrome: concepts and controversies. Lancet Neurol. (2013) 12:1180–8. 10.1016/S1474-4422(13)70215-1 24229616

[5] Jasti AK Selmi C Sarmiento-Monroy JC Vega DA Anaya JM Gershwin ME. Guillain-Barré syndrome: causes, immunopathogenic mechanisms and treatment. Expert Rev Clin Immunol. (2016) 12:1175–89. 10.1080/1744666X.2016.1193006 27292311

[6] Ozdemir HH. Analysis of the albumin level, neutrophil-lymphocyte ratio, and platelet-lymphocyte ratio in Guillain-Barré syndrome. Arq Neuropsiquiatr. (2016) 74:718–22. 10.1590/0004-282X20160132 27706420

[7] Rodríguez Y Rojas M Pacheco Y Acosta-Ampudia Y Ramírez-Santana C Monsalve DM . Guillain-barré syndrome, transverse myelitis and infectious diseases. Cell Mol Immunol. (2018) 15:547–62. 10.1038/cmi.2017.142 29375121 PMC6079071

[8] Olsson A Gustavsen S Gisselø Lauridsen K Chenoufi Hasselbalch I Sellebjerg F Bach Søndergaard H . Neutrophil-to-lymphocyte ratio and CRP as biomarkers in multiple sclerosis: a systematic review. Acta Neurol Scand. (2021) 143:577–86. 10.1111/ane.13401 33591593

[9] Leonhard SE Mandarakas MR Gondim FAA Bateman K Ferreira MLB Cornblath DR . Diagnosis and management of guillain-barré syndrome in ten steps. Nat Rev Neurol. (2019) 15:671–83. 10.1038/s41582-019-0250-9 31541214 PMC6821638

[10] Mayeux R. Biomarkers: potential uses and limitations. NeuroRx. (2004) 1:182–8. 10.1602/neurorx.1.2.182 15717018 PMC534923

[11] Mondello S Salama MM Mohamed WMY Kobeissy FH. Editorial: biomarkers in Neurology. Front Neurol. (2020) 11:190. 10.3389/fneur.2020.00190 32256443 PMC7093560

[12] Ballman KV. Biomarker: predictive or prognostic? J Clin Oncol. (2015) 33:3968–71. 10.1200/JCO.2015.63.3651 26392104

[13] Zeng Z Wang C Wang B Wang N Yang Y Guo S . Prediction of neutrophil-to-lymphocyte ratio in the diagnosis and progression of autoimmune encephalitis. Neurosci Lett. (2019) 694:129–35. 10.1016/j.neulet.2018.12.003 30521947

[14] Ji Z Liu G Guo J Zhang R Su Y Carvalho A . The neutrophil-to-lymphocyte ratio is an important indicator predicting in-hospital death in AMI patients. Front Cardiovasc Med. (2021) 8:706852. 10.3389/fcvm.2021.706852 34616780 PMC8488114

[15] Page MJ McKenzie JE Bossuyt PM Boutron I Hoffmann TC Mulrow CD . The PRISMA 2020 statement: an updated guideline for reporting systematic reviews. BMJ. (2021) 372:n71. 10.1136/bmj.n71 33782057 PMC8005924

[16] Hughes RA Newsom-Davis JM Perkin GD Pierce JM. Controlled trial prednisolone in acute polyneuropathy. Lancet. (1978) 2:750–3. 10.1016/S0140-6736(78)92644-2 80682

[17] van Koningsveld R Steyerberg EW Hughes RA Swan AV van Doorn PA Jacobs BC . clinical prognostic scoring system for Guillain-Barré syndrome. Lancet Neurol. (2007) 6:589–94. 10.1016/S1474-4422(07)70130-8 17537676

[18] Mendoza-Chuctaya G Montesinos-Segura R Agramonte-Vilca M Aguirre-Tenorio L. Características y prevalencia de partos domiciliarios en un distrito rural de la sierra del perú, 2015-2016. Rev Chil Obstet Ginecol. (2018) 83:377–85. 10.4067/s0717-7526201800040037727315006

[19] Ulloque-Badaracco JR Ivan Salas-Tello W Al-Kassab-Córdova A Alarcón-Braga EA Benites-Zapata VA Maguiña JL . Prognostic value of neutrophil-to-lymphocyte ratio in COVID-19 patients: a systematic review and meta-analysis. Int J Clin Pract. (2021) 75:e14596. 10.1111/ijcp.14596 34228867 PMC9614707

[20] Bedel C Korkut M Hospital R. The clinical significance of neutrophil lymphocyte ratio, monocyte lymphocyte ratio and platelet lymphocyte ratio in patients with guillain-barré syndrome. Haydarpaşa Numune Med J. (2021) 61:341–5. 10.14744/hnhj.2019.38233

[21] Ethemoglu O Calik M. Effect of serum inflammatory markers on the prognosis of adult and pediatric patients with Guillain-Barré syndrome. Neuropsychiatr Dis Treat. (2018) 14:1255–60. 10.2147/NDT.S162896 29805261 PMC5960237

[22] Geyik S Bozkurt H Neyal M Yigiter R Kuzudisli S Kul S . The clinical significance of the neutrophil-to-lymphocyte ratio in patients with Guillain-Barré syndrome independent of infection. Med Sci Discov. (2016) 3:305–11. 10.17546/msd.90383

[23] Hashim NA Mohamed WS Emad EM. Neutrophil–lymphocyte ratio and response to plasmapheresis in Guillain–Barré syndrome: a prospective observational study. Egypt J Neurol Psychiatry Neurosurg. (2020) 56:17. 10.1186/s41983-020-0154-z

[24] Huang Y Ying Z Quan W Xiang W Xie D Weng Y . The clinical significance of neutrophil-to-lymphocyte ratio and monocyte-to-lymphocyte ratio in Guillain-Barré syndrome. Int J Neurosci. (2018) 128:729–35. 10.1080/00207454.2017.1418342 29251087

[25] Gümüşyayla S Vural G. The predictive value of neutrophil-lymphocyte ratio in disability of gullain-barr? syndrome. Bakirköy Tip Dergisi. (2019) 15:187. 10.4274/BTDMJB.galenos.2019.20171214071335

[26] Sahin S Cinar N Karsidag S. Are cerebrospinal fluid protein levels and plasma neutrophil/lymphocyte ratio associated with prognosis of guillain barré syndrome? Neurol Int. (2017) 9:7032. 10.4081/ni.2017.7032 28713530 PMC5505084

[27] Tunç A. Early predictors of functional disability in Guillain-Barré Syndrome. Acta Neurol Belg. (2019) 119:555–9. 10.1007/s13760-019-01133-3 30963477

[28] Ning P Yang B Yang X Huang H Shen Q Zhao Q . Lymphocyte-based ratios for predicting respiratory failure in Guillain-Barré syndrome. J Neuroimmunol. (2021) 353:577504. 10.1016/j.jneuroim.2021.577504 33548620

[29] Guyatt GH Thorlund K Oxman AD Walter SD Patrick D Furukawa TA . GRADE guidelines: 13. preparing summary of findings tables and evidence profiles-continuous outcomes. J Clin Epidemiol. (2013) 66:173–83. 10.1016/j.jclinepi.2012.08.001 23116689

[30] Murad MH Mustafa RA Schünemann HJ Sultan S Santesso N. Rating the certainty in evidence in the absence of a single estimate of effect. Evid Based Med. (2017) 22:85–7. 10.1136/ebmed-2017-110668 28320705 PMC5502230

[31] Schünemann H Brozek J Guyatt G Oxman A. GRADE Handbook for Grading Quality of Evidence and Strength of Recommendation 2008. Available online at: https://gdt.gradepro.org/app/handbook/handbook.html

[32] Huguet A Hayden JA Stinson J McGrath PJ Chambers CT Tougas ME . Judging the quality of evidence in reviews of prognostic factor research: adapting the GRADE framework. Syst Rev. (2013) 2:71. 10.1186/2046-4053-2-71 24007720 PMC3930077

[33] Paliogiannis P Satta R Deligia G Farina G Bassu S Mangoni AA . Associations between the neutrophil-to-lymphocyte and the platelet-to-lymphocyte ratios and the presence and severity of psoriasis: a systematic review and meta-analysis. Clin Exp Med. (2019) 19:37–45. 10.1007/s10238-018-0538-x 30478648

[34] Erre GL Paliogiannis P Castagna F Mangoni AA Carru C Passiu G . Meta-analysis of neutrophil-to-lymphocyte and platelet-to-lymphocyte ratio in rheumatoid arthritis. Eur J Clin Invest. (2019) 49:e13037. 10.1111/eci.13037 30316204

[35] Ma L Zeng A Chen B Chen Y Zhou R. Neutrophil to lymphocyte ratio and platelet to lymphocyte ratio in patients with systemic lupus erythematosus and their correlation with activity: a meta-analysis. Int Immunopharmacol. (2019) 76:105949. 10.1016/j.intimp.2019.105949 31634817

[36] Hagen KM Ousman SS. The neuroimmunology of Guillain-Barré Syndrome and the potential role of an aging immune system. Front Aging Neurosci. (2021) 12:613628. 10.3389/fnagi.2020.613628 33584245 PMC7873882

[37] Guzel I Mungan S Oztekin ZN Ak F. Is there an association between the Expanded Disability Status Scale and inflammatory markers in multiple sclerosis? J Chin Med Assoc. (2016) 79:54–7. 10.1016/j.jcma.2015.08.010 26589195

[38] Morisaki T Goya T Ishimitsu T Torisu M. The increase of low density subpopulations and CD10 (CALLA) negative neutrophils in severely infected patients. Surg Today. (1992) 22:322–7. 10.1007/BF00308740 1392343

[39] Ng LG Ostuni R Hidalgo A. Heterogeneity of neutrophils. Nat Rev Immunol. (2019) 19:255–65. 10.1038/s41577-019-0141-8 30816340

[40] Rahman S Sagar D Hanna RN Lightfoot YL Mistry P Smith CK . Low-density granulocytes activate T cells and demonstrate a non-suppressive role in systemic lupus erythematosus. Ann Rheum Dis. (2019) 78:957–66. 10.1136/annrheumdis-2018-214620 31040119 PMC6585283

[41] Ren K Yang A Lu J Zhao D Bai M Ding J . Association between serum low-density neutrophils and acute-onset and recurrent Guillain-Barré syndrome. Brain Behav. (2022) 12:e2456. 10.1002/brb3.2456 34894104 PMC8785626

[42] Forget P Khalifa C Defour JP Latinne D Van Pel MC De Kock M. What is the normal value of the neutrophil-to-lymphocyte ratio? BMC Res Notes. (2017) 10:12. 10.1186/s13104-016-2335-5 28057051 PMC5217256

[43] Moisa E Corneci D Negoita S Filimon CR Serbu A Negutu MI . Dynamic changes of the neutrophil-to-lymphocyte ratio, systemic inflammation index, and derived neutrophil-to-lymphocyte ratio independently predict invasive mechanical ventilation need and death in critically Ill COVID-19 patients. Biomedicines. (2021) 9:1656. 10.3390/biomedicines9111656 34829883 PMC8615772

[44] Nair PR Maitra S Ray BR Anand RK Baidya DK Subramaniam R. Neutrophil-to-lymphocyte ratio and platelet-to-lymphocyte ratio as predictors of the early requirement of mechanical ventilation in COVID-19 patients. Indian J Crit Care Med. (2020) 24:1143–4. 10.5005/jp-journals-10071-23663 33384528 PMC7751036

[45] Yang AP Liu JP Tao WQ Li HM. The diagnostic and predictive role of NLR, d-NLR and PLR in COVID-19 patients. Int Immunopharmacol. (2020) 84:106504. 10.1016/j.intimp.2020.106504 32304994 PMC7152924

[46] Liu JA Yu J Cheung CW. Immune actions on the peripheral nervous system in pain. Int J Mol Sci. (2021) 22:1448. 10.3390/ijms22031448 33535595 PMC7867183

[47] Fokke C van den Berg B Drenthen J Walgaard C van Doorn PA Jacobs BC. Diagnosis of Guillain-Barré syndrome and validation of Brighton criteria. Brain. (2014) 137:33–43. 10.1093/brain/awt285 24163275

[48] Arami MA Yazdchi M Khandaghi R. Epidemiology and characteristics of Guillain-Barré syndrome in the northwest of Iran. Ann Saudi Med. (2006) 26:22–7. 10.5144/0256-4947.2006.22 16521871 PMC6078541

[49] Tian J Cao C Li T Zhang K Li P Liu Y . Electrophysiological subtypes and prognostic factors of Guillain-Barre Syndrome in Northern China. Front Neurol. (2019) 10:714. 10.3389/fneur.2019.00714 31333568 PMC6614537

[50] Doets AY Verboon C van den Berg B Harbo T Cornblath DR Willison HJ . Regional variation of Guillain-Barré syndrome. Brain. (2018) 141:2866–77. 10.1093/brain/awy232 30247567

[51] Doets AY Lingsma HF Walgaard C Islam B Papri N Davidson A . Predicting outcome in Guillain-Barré Syndrome: international validation of the modified erasmus GBS outcome score. Neurology. (2022) 98:e518–32. 10.1212/WNL.0000000000013139 34937789 PMC8826467

[52] Doets AY Walgaard C Lingsma HF Islam B Papri N Yamagishi Y . International validation of the erasmus Guillain-Barré Syndrome respiratory insufficiency score. Ann Neurol. (2022) 91:521–31. 10.1002/ana.26312 35106830 PMC9306880

[53] Malaga M Rodriguez-Calienes A Marquez-Nakamatsu A Recuay K Merzthal L Bustamante-Paytan D . Predicting mechanical ventilation using the egris in Guillain-Barré Syndrome in a Latin American country. Neurocrit Care. (2021) 35:775–82. 10.1007/s12028-021-01218-z 34021483

[54] Zahorec R. Neutrophil-to-lymphocyte ratio, past, present and future perspectives. Bratisl Lek Listy. (2021) 122:474–88. 10.4149/BLL_2021_078 34161115

[55] van Koningsveld R Schmitz PI Meché FG Visser LH Meulstee J van Doorn PA. Effect of methylprednisolone when added to standard treatment with intravenous immunoglobulin for Guillain-Barré syndrome: randomised trial. Lancet. (2004) 363:192–6. 10.1016/S0140-6736(03)15324-X 14738791

[56] Walgaard C Jacobs BC Lingsma HF Steyerberg EW van den Berg B Doets AY . Second intravenous immunoglobulin dose in patients with Guillain-Barré syndrome with poor prognosis (SID-GBS): a double-blind, randomised, placebo-controlled trial. Lancet Neurol. (2021) 20:275–83. 10.1016/S1474-4422(20)30494-4 33743237

[57] Orlikowski D Sharshar T Porcher R Annane D Raphael JC Clair B. Prognosis and risk factors of early onset pneumonia in ventilated patients with Guillain-Barré syndrome. Intensive Care Med. (2006) 32:1962–9. 10.1007/s00134-006-0332-1 17019557

[58] Péter M Török W Petrovics-Balog A Vígh L Vécsei L Balogh G. Cerebrospinal fluid lipidomic biomarker signatures of demyelination for multiple sclerosis and Guillain-Barré syndrome. Sci Rep. (2020) 10:18380. 10.1038/s41598-020-75502-x 33110173 PMC7592055

[59] Breville G Lascano AM Roux-Lombard P Lalive PH. IL-8 as a potential biomarker in Guillain-Barre Syndrome. Eur Cytokine Netw. (2019) 30:130–4. 10.1684/ecn.2019.0436 32096474

