# Supplementary material for: The neutrophil-to-lymphocyte ratio as a prognostic biomarker in Guillain-Barre syndrome: a systematic review with meta-analysis
Source: Front Neurol. 2023 Jun 2;14:1153690. doi: 10.3389/fneur.2023.1153690 (PMC10272825; doi:10.3389/fneur.2023.1153690)
Supplement: Supplementary file 1 [file Data_Sheet_1.pdf]

Supplementary material

Supplementary material 1. Search strategy

Database	Search strategy	Results
Pubmed	#1 Guillain-Barre Syndrome<br>(Guillain-Barre Syndrome[MH] OR Guillain*[all fields] OR "gullain*"[all fields] OR "GBS"[all<br>fields] OR "Acute neuropath*" [all fields] OR "Acute polyneuritis" [all fields] OR Miller Fisher<br>Syndrome [MH] OR “Miller Fisher”[all fields] OR “Miller-Fisher” [all fields] OR “Fisher<br>Syndr*”[all fields] OR polyradiculoneuropathy [MH] OR polyradiculoneuropathy, chronic<br>inflammatory demyelinating [MH] OR polyradiculo*[all fields] OR polyneuropath*[all fields] OR<br>cidp[all fields])<br><br>#2 Neutrophil-to-lymphocyte ratio<br>("neutrophil to lymphoc*" [all fields] OR “neutrophil-to-lymphoc*”[all fields] OR<br>“neutrophil/lymphoc*” OR "NLR" [all fields] OR "neutrophil lymphoc*" [all fields] OR<br>"granulocyte lymphoc*" [all fields] OR “granulocyte to lymphoc*” [all fields] OR “granulocyteto-lymphoc*”[all fields] OR “granulocyte/lymphoc*” OR "GLR" [all fields])<br><br>#3: #1 AND #2	27
Scopus	#1 Guillain-Barre Syndrome<br>ALL(Guillain* OR GBS OR "Acute neuropath*" OR "Acute polyneuritis" OR “Miller Fisher” OR<br>“Miller-Fisher” OR “Fisher Syndr*” OR polyradiculo* OR polyneuropath* OR CIDP)<br><br>#2 Neutrophil-to-lymphocyte ratio<br>ALL("neutrophil to lymphoc*" OR “neutrophil-to-lymphoc*” OR “neutrophil/lymphoc*” OR<br>"NLR" OR "neutrophil lymphoc*" OR "granulocyte lymphoc*" OR “granulocyte to lymphoc*”<br>OR “granulocyte-to-lymphoc*” OR “granulocyte/lymphoc*” OR "GLR")<br><br>#3: #1 AND #2	531

WOS	#1 Guillain-Barre Syndrome<br>TI=(Guillain* OR GBS OR "Acute neuropath*" OR "Acute polyneuritis" OR “Miller Fisher” OR<br>“Miller-Fisher” OR “Fisher Syndr*” OR polyradiculo* OR polyneuropath* OR cidp) OR<br>TS=(Guillain* OR GBS OR "Acute neuropath*" OR "Acute polyneuritis" OR “Miller Fisher” OR<br>“Miller-Fisher” OR “Fisher Syndr*” OR polyradiculo* OR polyneuropath* OR cidp) OR<br>KP=(Guillain* OR GBS OR "Acute neuropath*" OR "Acute polyneuritis" OR “Miller Fisher” OR<br>“Miller-Fisher” OR “Fisher Syndr*” OR polyradiculo* OR polyneuropath*) OR AK=(Guillain* OR<br>GBS OR "Acute neuropath*" OR "Acute polyneuritis" OR “Miller Fisher” OR “Miller-Fisher” OR<br>“Fisher Syndr*” OR polyradiculo* OR polyneuropath* OR cidp) OR AB=(Guillain* OR GBS OR<br>"Acute neuropath*" OR "Acute polyneuritis" OR “Miller Fisher” OR “Miller-Fisher” OR “Fisher<br>Syndr*” OR polyradiculo* OR polyneuropath* OR cidp)<br><br>#2 Neutrophil-to-lymphocyte ratio<br>TI=("neutrophil to lymphoc*" OR “neutrophil-to-lymphoc*” OR “neutrophil/lymphoc*” OR<br>"NLR" OR "neutrophil lymphoc*" OR "granulocyte lymphoc*" OR “granulocyte to lymphoc*”<br>OR “granulocyte-to-lymphoc*” OR “granulocyte/lymphoc*” OR "GLR") OR TS=("neutrophil to<br>lymphoc*" OR “neutrophil-to-lymphoc*” OR “neutrophil/lymphoc*” OR "NLR" OR "neutrophil<br>lymphoc*" OR "granulocyte lymphoc*" OR “granulocyte to lymphoc*” OR “granulocyte-tolymphoc*” OR “granulocyte/lymphoc*” OR "GLR") OR KP=("neutrophil to lymphoc*" OR<br>“neutrophil-to-lymphoc*” OR “neutrophil/lymphoc*” OR "NLR" OR "neutrophil lymphoc*" OR<br>"granulocyte lymphoc*" OR “granulocyte to lymphoc*” OR “granulocyte-to-lymphoc*” OR<br>“granulocyte/lymphoc*” OR "GLR") OR AK=("neutrophil to lymphoc*" OR “neutrophil-tolymphoc*” OR “neutrophil/lymphoc*” OR "NLR" OR "neutrophil lymphoc*" OR "granulocyte<br>lymphoc*" OR “granulocyte to lymphoc*” OR “granulocyte-to-lymphoc*” OR<br>“granulocyte/lymphoc*” OR "GLR") OR AB=("neutrophil to lymphoc*" OR “neutrophil-tolymphoc*” OR “neutrophil/lymphoc*” OR "NLR" OR "neutrophil lymphoc*" OR "granulocyte	37

	lymphoc*" OR “granulocyte to lymphoc*” OR “granulocyte-to-lymphoc*” OR<br>“granulocyte/lymphoc*” OR "GLR")<br><br>#3: #1 AND #2	
Ovid Medline	#1: Guillain-Barre Syndrome/<br>#2: Miller Fisher Syndrome/<br>#3: polyradiculoneuropathy/<br>#4: polyradiculoneuropathy, chronic inflammatory demyelinating/<br>#5: (Guillain* OR GBS OR Acute neuropath* OR Acute polyneuritis OR Miller Fisher OR MillerFisher OR Fisher Syndr* OR polyradiculo* OR polyneuropath*).mp<br>#6: #1 OR #2 OR #3 OR #4 OR #5<br>#7: (neutrophil to lymphoc* OR neutrophil-to-lymphoc* OR NLR OR neutrophil lymphoc* OR<br>granulocyte lymphoc* OR granulocyte to lymphoc* OR granulocyte-to-lymphoc* OR GLR).mp<br><br>#8: #6 AND #7	26
Embase	#1 Guillain-Barre Syndrome<br>('guillain barre syndrome'/exp OR 'fisher syndrome' OR 'guillain barre' OR 'guillain barre<br>disease' OR 'guillain barre polyradiculitis' OR 'guillain barre polyradiculoneuritis' OR 'guillain<br>barre syndrome' OR 'guillain-barre syndrome' OR 'landry guillain barre strohl syndrome' OR<br>'landry guillain barre syndrome' OR 'landry paralysis' OR 'landry syndrome' OR 'miller fisher<br>syndrome' OR 'acute febrile polyneuritis' OR 'acute postinfective polyradiculoneuropathy' OR<br>'infectious neuronitis' OR 'inflammatory acute polyradiculoneuropathy' OR 'polyradiculoneuritis guillain-barre' OR 'polyradiculoneuropathy, acute postinfective' OR<br>'polyradiculoneuropathy, inflammatory acute' OR gbs)<br><br>#2 Neutrophil-to-lymphocyte ratio<br>('neutrophil lymphocyte ratio'/exp<br>OR 'nlr (lymphocyte)' OR 'neutrophil lymphocyte ratio' OR 'neutrophil to lymphocyte ratio' OR<br>'neutrophil/lymphocyte ratio' OR nlr OR glr OR 'neutrophil to lymphocyte' OR 'granulocyte<br>lymphocyte')<br><br>#3: #1 AND #2	23
LILACS	#1 Guillain-Barre Syndrome	1

	((neutrofilo-linfocito) OR (neutrophile-linfocito) OR (neutrofilo/linfocito) OR<br>(neutrophil/lymphocyte) OR (neutrophil-to-lymphocyte))<br><br>#2 Neutrophil-to-lymphocyte ratio<br>(("Guillain Barre") OR ("GuillainBarre") OR ("Guillain Barré") OR ("Guillain-Barré") OR (SGB) OR (“GBS”) OR (“Miller Fisher”) OR (“Miller-Fisher”))<br><br>#3: #1 AND #2	
Google Scholar	#1 Guillain-Barre Syndrome<br>(guillain*)<br><br>#2 Neutrophil-to-lymphocyte ratio<br>("neutrophil to lymphoc*" OR “neutrophil-to-lymphoc*” OR nlr)<br><br>#3: #1 AND #2	100

Supplementary material 2. Excluded studies

Autor-Año	Título	Razones de exclusión
Aziz -2021	Association of different parameters of complete blood count with severity and prognosis of Guillain-Barre Syndrome	Otro factor de exposición
Wu -2021	Increased systemic immune-inflammation index can predict respiratory failure in patients with Guillain-Barré syndrome	Otro factor de exposición
Hasanbaş -2020	The relationship between clinical and biological biomarkers with disease outcome In Guillain-Barré Syndrome	Resumen de Congreso
Juananda-2021	Comparison of neutrophil to lymphocyte ratio and platelet to lymphocyte ratio before and after plasma exchange treatment in Guillain-barre syndrome patients	Resumen de Congreso
Tiwari-2021	Clinical Profile and Predictors of Mechanical Ventilation in Guillain-Barre Syndrome in North Indian Children	Otra población
Hüner-2018	Association of Neutrophil/Lymphocyte Ratio With Intravenous Immunoglobulin Treatment in Children With Guillain-Barre Syndrome	Otra población
Zhang Weiwei -2020	Correlation Analysis of Peripheral Blood Inflammation Indicators and Guillain-Barré Syndrome	Sin acceso a texto completo
Chen Congyan -2019	Analysis of neutrophil-lymphocyte ratio and serum albumin in patients with Guillain-Barré syndrome	Sin acceso a texto completo

Supplementary material 3. Leave-one-out sensitivity analysis

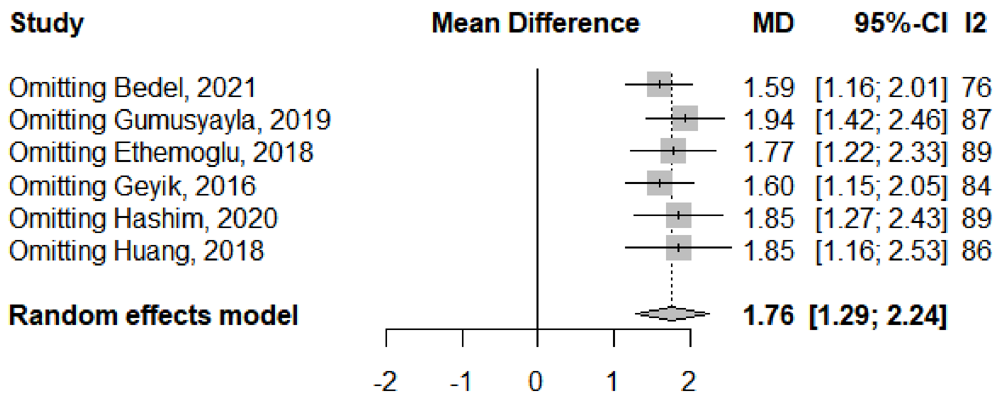

Supplementary material 4. Sensitive analysis including only studies with case-control match

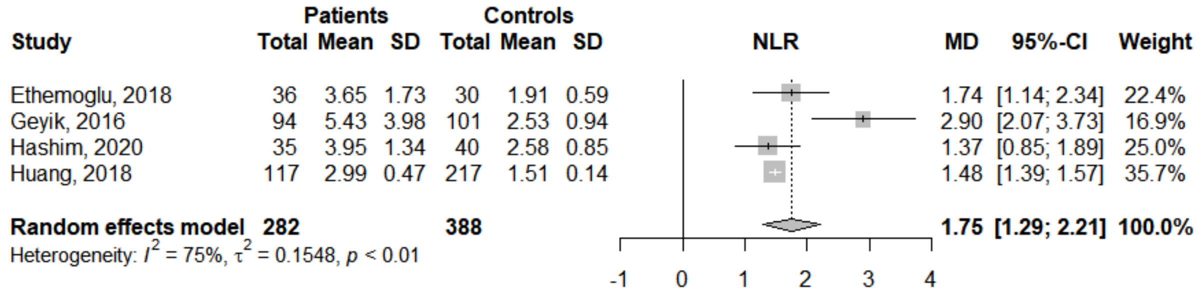

Supplementary material 5. NEWCASTLE-OTTAWA QUALITY ASSESSMENT SCALE FOR INCLUDED STUDIES

NEWCASTLE - OTTAWA QUALITY ASSESSMENT SCALE FOR COHORT STUDIES

STUDY	**SELECTION**				**COMPARABILITY**	**OUTCOME**				
	**Representativeness of the exposed cohort**	**Sample size**	**Ascertainment of exposure**	**Definition of the cohorts**	**Comparability of Cohorts on the Basis of the Design or Analysis (Maximum: ☆☆)**	**Assessment of outcome**	**Was follow-up long enough for outcomes to occur?**	**Adequacy of follow up of cohorts?**	**SCORE**	**Risk of bias**
Ethemoglu et.al.	☆		☆	☆	☆	☆	☆	☆	*7*	Low Risk of bias
Geyik et.al	☆		☆	☆		☆	☆	☆	*6*	Low Risk of bias
Ali Hashim et.al			☆	☆		☆	☆	☆	*5*	Moderate Risk of bias
Huang et.al	☆		☆	☆	☆	☆	☆	☆	*7*	Low Risk of bias
Gumusyayla et.al			☆			☆	☆		*3*	Moderate Risk of bias
Sahin et.al			☆	☆	☆	☆	☆	☆	*6*	Low Risk of bias
Tunç et.al	☆		☆	☆		☆	☆	☆	*6*	Low Risk of bias
Ning et.al	☆		☆	☆	☆	☆	☆	☆	*7*	Low Risk of bias
Ozdemir et.al	☆		☆	☆		☆	☆	☆	*6*	Low Risk of bias

NEWCASTLE - OTTAWA QUALITY ASSESSMENT SCALE FOR CASE-CONTROL STUDIES

STUDY	**SELECTION**	**COMPARABILITY**	**OUTCOME**

	Is the case definition adequate?	Representativeness of the cases	Selection of Controls	Definition of Controls	Comparability of cases and controls on the basis of the design or analysis (Maximum: ☆☆)	Assessment of exposure	Method of ascertainment	Non-response rate	SCORE	Evidence quality
Bedel et.al	☆	☆		☆	☆	☆	☆		6	Low Risk of bias
